# Special Issue “Molecular Research in Bamboo, Tree, Grass, and Other Forest Products”

**DOI:** 10.3390/ijms27125248

**Published:** 2026-06-10

**Authors:** Huayu Sun

**Affiliations:** 1 Institute of Gene Science and Industrialization for Bamboo and Rattan Resources, International Centre for Bamboo and Rattan, Beijing 100102, China; sunhy@icbr.ac.cn; 2 Key Laboratory of National Forestry and Grassland Administration on Bamboo & Rattan, Beijing 100102, China

As the Earth’s resources are increasingly being depleted, fossil resources such as oil and natural gas are becoming scarcer, making renewable resources the focus of this Editorial [1]. Bamboo, trees, grasses, and other forest resources are all renewable. They not only play a vital role in sustaining the Earth’s environment but also serve as crucial substitutes for plastics, metals, and other materials in the production of chemical products. In an era defined by the dual imperatives of environmental sustainability and climate resilience, forest products—encompassing bamboo, trees, grasses, and their myriad derivatives—have emerged as indispensable pillars for a green future [2,3,4]. The call for this Special Issue rightly emphasized their critical role in providing renewable materials, enhancing biodiversity, and driving eco-innovation. To transform this potential into reality, moving from phenomenological observation to mechanistic understanding is paramount. Molecular biology research serves as the essential lens for deciphering the genetic and biochemical codes governing the growth, adaptation, and utility of vital biological resources. By closely studying biopolymers—DNAs, RNAs, and proteins—and their interactions, this field aims to unravel the intricate mechanisms of life [5].

This Special Issue, titled “Molecular Research in Bamboo, Tree, Grass, and Other Forest Products”, successfully captures the vibrant activity in this field. We are delighted to present a curated collection of eight original research articles that collectively address the core themes outlined in our initial call for papers, from foundational genetics to applied sustainability (Table 1). These contributions not only report significant discoveries but also weave together a narrative of how molecular science is actively shaping the future of forest-based solutions.

Forest biodiversity experiments investigate how species diversity influences ecosystem functioning, particularly forest productivity. Understanding these dynamics can help bridge biodiversity–ecosystem function research, with practical goals for ecological restoration and forest management within the global biodiversity framework [6].

To grasp the resilience of forest ecosystems, it is essential to study both plants and their associated biotic partners. Forests across nearly all regions worldwide are increasingly impacted by invasions of non-native insects [7]. Opening this Special Issue, the study by Wieczorek et al. (Contribution 1), employs mitochondrial COI gene analysis to reconstruct the invasion history of an aphid. It reveals a dramatic genetic bottleneck post-introduction, offering a compelling molecular forensics case study on invasion dynamics and biosecurity.

In arid and semi-arid regions, rising soil salinization poses a major threat to global agricultural productivity and food security [8]. Developing salinity-tolerant crop varieties through sustainable strategies is critical to mitigating the adverse effects of salt stress on plant growth [9]. Complementing this organism-level interaction, the research by Qi et al. (Contribution 2) delves into molecular-level stress resistance in a turfgrass, identifying key hexokinase genes that respond dynamically to salt stress and providing candidate genes for breeding salinity-tolerant varieties. Grasses—encompassing turf and forage species—cover a vast portion of the Earth’s terrestrial surface. They play a pivotal ecological and economic role in soil conservation, water management, livestock forage production, and carbon sequestration. Consequently, research into their salinity tolerance and other stress responses is of significant scientific and practical value. Looking forward, emerging technologies such as the CRISPR-Cas system hold substantial promise for unlocking the full genetic potential of grasses, enabling targeted improvements in the resilience, yield, and quality of turf and forage species [10,11].

Collectively, research into both biotic stress and environmental adaptation in plants will deepen our understanding of the evolution and physiology of key species—from bamboo and trees to grasses and other forest products. This knowledge forms the essential foundation for future targeted genetic improvements, ultimately supporting enhanced resilience and productivity in the face of environmental challenges.

The extensive genetic diversity present both among and within thousands of forest tree species worldwide harbors valuable traits of economic, ecological, scientific, and social importance for humanity. Unlocking this genetic potential is essential to optimize the utilization of economically valuable tree species and to develop improved genotypes that maximize productivity within specific spatiotemporal constraints [12]. The genetic improvement of forest species relies on a deep understanding of their genomes and the regulators of key traits. Forest biotechnology is rapidly evolving from conventional breeding toward molecular design, enabling the development of genetically modified trees with enhanced traits such as faster growth, stress resilience, and superior wood properties [13].

Several papers in this issue enrich this molecular biology and genetics foundation. Lozano-Puentes et al. (Contribution 3) (“Assessing Genetic Variation in *Guadua angustifolia* Through RAD-Seq Analysis”) provide a comprehensive analysis of genetic diversity within this economically vital bamboo, identifying populations with unique genetic structures crucial for conservation and breeding. Their identification of populations with distinct genetic architecture offers vital insights for conservation strategies and breeding programs, underscoring the value of natural genetic diversity as a fundamental resource for both scientific inquiry and applied improvement [14,15].

Moving from broad genetic surveys to the dissection of specific complex traits, other studies employ genome-wide analyses to pinpoint key regulatory components. The flower color has drawn extensive attention in rapeseed breeding for its ornamental value [16]. Liu et al. (Contribution 4) (“Genome-Wide Characterization of WRKY Gene Family in *Camellia chekiangoleosa* Identifies Potential Regulatory Components in Pigment Biosynthesis Pathways”) characterize the *WRKY* transcription factor family, pinpointing members likely involved in flower color regulation. Similarly, Chen et al. (Contribution 5) (“Genome-Wide Identification of 109 NAC Genes and Dynamic Expression Profiles Under Cold Stress in *Madhuca longifolia*”) systematically uncover the *NAC* gene family in an oil tree, revealing specific genes that rapidly respond to cold, offering targets for enhancing frost resilience. Meanwhile, Li et al. (Contribution 6) (“Identification of miRNAs and Their Targets in *Cunninghamia lanceolata* Under Low Phosphorus Stress”) tackle nutrient stress at the post-transcriptional level, identifying key microRNAs and target genes that orchestrate the adaptive response to phosphorus deficiency in Chinese fir.

Decoding the genetic variation, distribution of gene families, and expression patterns of coding and non-coding genes in response to natural stress environments in bamboo, trees, grasses, and other forest products will lead to a clearer understanding of these species and support more precise breeding efforts.

Green chemistry is an interdisciplinary field focused on minimizing hazardous substances in chemical processes and promoting sustainable alternatives to conventional products and methods. It plays a fundamental role as a strategic approach for advancing sustainable development and supporting the achievement of the Sustainable Development Goals outlined in the 2030 Agenda [17,18]. The ultimate test of molecular knowledge lies in its application towards sustainable materials and green chemistry. This issue features pioneering work that connects gene function and physiology to tangible traits and future applications.

The study by Huang et al. (Contribution 7) (“Cytological, Physiological, and Transcriptome Analysis of Leaf-Yellowing Mutant in *Camellia chekiangoleosa*”) provides a holistic, multi-omics analysis of a chlorophyll-deficient mutant, elucidating the physiological consequences and transcriptional reprogramming underlying leaf coloration—a trait of both ecological and ornamental significance.

The comprehensive review by Di et al. (Contribution 8) (“Development and Future Prospects of Bamboo Gene Science”) synthesizes the rapid advancements in bamboo genomics and biotechnology. Bamboo exhibits an extraordinary growth capacity, with certain species growing over 1 m per day during peak phases, reaching full height within 1.5 months. Fiber production typically begins within 1–2 years, while harvest-ready culms are available in 3–5 years [19,20]. It charts a course for leveraging this knowledge to overcome long-standing challenges, such as flowering control and engineering bamboo for enhanced material properties, directly supporting global initiatives like “Bamboo as a Substitute for Plastic.”

The replacement of fossil-based resources with renewable alternatives—including bamboo, trees, grasses, and other forest-derived products—represents an unstoppable trend. Decoding and effectively utilizing these renewable resources to progressively displace non-renewable energy sources is essential for achieving truly sustainable development.

This collection of eight studies traces a coherent and impactful scientific continuum. The journey begins with ecological surveillance and genetic resource mapping—tracking invasive species (Contribution 1) and mapping genetic diversity (Contribution 3)—before moving on to dissecting gene families for stress and pigment regulation (Contribution 2; Contribution 4; Contribution 5; Contribution 6) and bridging phenotypes and molecular mechanisms (Contribution 7), culminating in a roadmap for translational science (Contribution 8). Together, these works decisively affirm that molecular research has moved from a peripheral to central position in the sustainable management and value creation for forest resources (Figure 1).

Looking ahead, the path forward is one of integration and translation. The challenges and opportunities illuminated across these papers demand convergent, interdisciplinary solutions. Key frontiers include the following:

Decoding Complex Traits: Bridging genotype to phenotype for adaptive and economic traits remains a fundamental challenge. While genomic associations identify candidate regions, there is a pressing need to elucidate the causal molecular mechanisms. Integrating high-resolution tools like single-cell omics with advanced computational models offers a powerful pathway to unravel the dynamic interplay between genes and traits, unlocking new dimensions of biological understanding [21].

Enabling Genetic Innovation: Advancing genetic transformation and regeneration protocols is crucial for functional genomics and trait engineering. Plant transformation is indispensable for introducing novel characteristics or modifying existing ones [22,23]. Furthermore, regeneration—a foundational biological process mirrored in techniques from cutting propagation to tissue culture—is vital for plant propagation and biotechnology [24]. Enhancing these technologies is key to developing climate-resilient crops and sustainable forestry solutions.

Scaling Discovery to Application: Translating laboratory breakthroughs to field and industrial scales requires us to address the automation and standardization of science. Laboratory automation accelerates discovery but introduces challenges in flexible autonomy, reproducibility, throughput, and defining the evolving role of researchers [25]. Overcoming these hurdles is essential for the robust deployment of new technologies.

We envision a future where systems biology, genome editing, and synthetic biology converge to enable the rational design of forest plants—optimized for enhanced carbon sequestration, greater resilience to environmental stresses, and bespoke material properties.

We are deeply grateful to all the authors for their outstanding contributions, which form the core of this Special Issue. We also extend our sincere thanks to the numerous expert reviewers, whose rigorous evaluations ensured the quality of this collection, and the editorial staff for their unwavering support. It is our hope that this collection will serve as a valuable reference and a catalyst for further interdisciplinary research, inspiring continued innovation to harness the molecular wisdom of forests for the benefit of our planet and society.

## Figures and Tables

**Figure 1:**
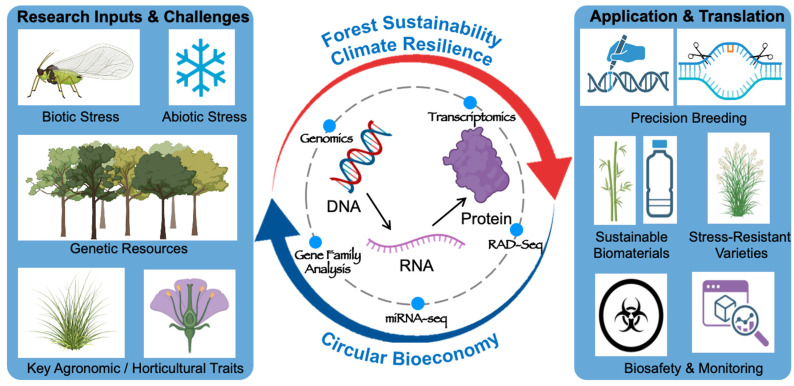
A translational research framework for forest plants.

**Table 1:** Overview of articles included in this Special Issue.

Article	Key Research Theme	Study System/Species	Core Methodology/Technology	Main Findings/Contributions
Contribution 1	Ecological Interactions & Invasion Genetics	Bamboo aphid (*Takecallis taiwanus*) and arrow bamboo (*Fargesia spathacea*)	Mitochondrial COI gene sequencing, population genetic analysis	Revealed genetic bottlenecks and invasion pathways underlying a recent pest outbreak.
Contribution 2	Abiotic Stress Response	Buffalograss (*Bouteloua dactyloides*)	Genome-wide gene family identification and expression profiling	Identified key HXK genes responsive to salt stress.
Contribution 3	Genetic Diversity Assessment	Guadua bamboo (*Guadua angustifolia*)	RAD-Seq (Restriction-site Associated DNA Sequencing)	Constructed a high-resolution map of genetic diversity and population structure for this economically important bamboo species.
Contribution 4	Transcriptional Regulation & Trait Elucidation	Zhejiang red camellia (*Camellia chekiangoleosa*)	Genome-wide WRKY family analysis, expression–phenotype association	Uncovered candidate WRKY transcription factors potentially regulating floral pigment biosynthesis.
Contribution 5	Transcriptional Regulation & Stress Resistance	Long-leaf madhuca (*Madhuca longifolia*)	Genome-wide NAC family analysis, cold stress expression profiling	Pinpointed core NAC genes that are rapidly induced by cold stress.
Contribution 6	Epigenetics & Nutrient Stress	Chinese fir (*Cunninghamia lanceolata*)	Small RNA sequencing (sRNA-seq) and degradome sequencing	Systematically deciphered the miRNA-target gene regulatory network under phosphate deficiency.
Contribution 7	Multi-omics Analysis of a Mutant	Zhejiang red camellia (*Camellia chekiangoleosa*) chlorophyll-deficient mutant	Integrated cytological, physiological, and transcriptomic analysis	Elucidated the physiological and molecular mechanisms driving leaf chlorosis in this ornamental mutant.
Contribution 8	Synthesis Review & Future Perspectives	Bamboo species	Literature review and prospective analysis	Synthesized advances in bamboo genomics and proposed future translational directions, including applications as sustainable alternatives to plastics.
